# Characterization of the Proteome of Cytoplasmic Lipid Droplets in Mouse Enterocytes after a Dietary Fat Challenge

**DOI:** 10.1371/journal.pone.0126823

**Published:** 2015-05-18

**Authors:** Theresa D’Aquila, Devika Sirohi, Jeffrey M. Grabowski, Victoria E. Hedrick, Lake N. Paul, Andrew S. Greenberg, Richard J. Kuhn, Kimberly K. Buhman

**Affiliations:** 1 Department of Nutrition Science, Purdue University, West Lafayette, Indiana, United States of America; 2 Department of Biological Sciences, Purdue University, West Lafayette, Indiana, United States of America; 3 Bindley Bioscience Center, Purdue University, West Lafayette, Indiana, United States of America; 4 Department of Entomology, Purdue University, West Lafayette, Indiana, United States of America; 5 Jean Mayer USDA Human Nutrition Research Center on Aging, Tufts University, Boston, Massachusetts, United States of America; National Institute of Agronomic Research, FRANCE

## Abstract

Dietary fat absorption by the small intestine is a multistep process that regulates the uptake and delivery of essential nutrients and energy. One step of this process is the temporary storage of dietary fat in cytoplasmic lipid droplets (CLDs). The storage and mobilization of dietary fat is thought to be regulated by proteins that associate with the CLD; however, mechanistic details of this process are currently unknown. In this study we analyzed the proteome of CLDs isolated from enterocytes harvested from the small intestine of mice following a dietary fat challenge. In this analysis we identified 181 proteins associated with the CLD fraction, of which 37 are associated with known lipid related metabolic pathways. We confirmed the localization of several of these proteins on or around the CLD through confocal and electron microscopy, including perilipin 3, apolipoprotein A-IV, and acyl-CoA synthetase long-chain family member 5. The identification of the enterocyte CLD proteome provides new insight into potential regulators of CLD metabolism and the process of dietary fat absorption.

## Introduction

Dietary fat is the most energy dense macronutrient consumed and is required for the absorption of essential fatty acids and other lipophilic nutrients including fat soluble vitamins. However, when present in excess, dietary fat increases the risk for chronic diseases such as cardiovascular disease and obesity [1–4]. Therefore, understanding the regulators of dietary fat absorption and metabolism is important for both the promotion of health and prevention of disease.

Dietary fat absorption by the small intestine is a multistep process. Triacylglycerol (TAG) is hydrolyzed by pancreatic lipase in the intestinal lumen producing monoacylglycerol and free fatty acids. These digestive products are taken up by the absorptive cells of the intestine, enterocytes, where they are rapidly resynthesized to TAG. The TAG is then packaged in the core of a chylomicron for systemic delivery of nutrients throughout the body [3, 5, 6]. Alternatively, when fatty acids are present in excess, the newly synthesized TAG may be incorporated into cytoplasmic lipid droplets (CLDs) within enterocytes. The size and number of CLDs within enterocytes increases and then decreases after consumption of dietary fat [7]; however, the factors that regulate CLD synthesis and catabolism within enterocytes are relatively unknown. The partitioning of TAG into chylomicrons or CLDs is important for determining the amount and rate of fatty acids delivered systemically. Therefore, identification of factors that regulate enterocyte CLD metabolism is important for understanding the overall process of dietary fat absorption.

Proteins that associate with CLDs in various cell types have been shown to regulate the synthesis and catabolism of CLDs [8–11]. Recently, our laboratory identified two CLD associated proteins, perilipin 2 (Plin2) and perilipin 3 (Plin3), on CLDs within enterocytes after a dietary fat challenge [12]. These well-established CLD associated proteins are thought to regulate lipolysis. In addition, mice deficient in mediators of lipolysis, including adipose triglyceride lipase [13] and abhydrolase domain containing 5 [14], have altered catabolism of CLDs in enterocytes. These results strongly suggest CLD associated proteins regulate the synthesis and catabolism of CLDs in enterocytes. The identification of additional CLD associated proteins within enterocytes has the potential to establish novel mediators of dietary fat absorption.

The objective of this study was to identify CLD associated proteins in enterocytes after a dietary fat challenge. To fulfill this objective, we isolated CLDs from the small intestine of mice two hours after an oil bolus and identified proteins involved in the regulation of lipid trafficking and metabolism using a global proteomic approach. We further validated the presence of selected proteins on or around CLDs by confocal and immunoelectron microscopy.

## Materials and Methods

### Ethics statement

We conducted the study in strict accordance with the recommendations in the Guide for the Care and Use of Laboratory Animals of the National Institute of Health. The protocol was approved by the Purdue Animal Care and Use Committee (PACUC# 1111000154). All effort was made to minimize pain and suffering of mice and the number of mice used.

### Mice

C57BL/6 male mice from an in-house breeding colony were used for this study. The mice were maintained on a chow diet (PicoLab 5053, Lab Diets, Richmond, IN, USA) that consisted of 62.1% of calories from carbohydrate (starch), 24.7% from protein, and 13.2% from fat. The mice were housed in a temperature and humidity controlled facility with a 12 hour light/dark cycle (6AM/6PM) with ad libitum access to food and water.

### Mouse procedure for lipid droplet isolation and proteomics analysis

Four, 5 month old male C57BL/6 mice were fasted for 4 hours at the beginning of the light cycle. An oral gavage of 200 μl olive oil was administered and two hours after the oil bolus, the mice were euthanized via CO_2_ asphyxiation. The small intestine was excised and divided into five equal length segments in relation to the stomach and labeled sections 1–5. Segments 2 and 3, representing the jejunum, were used for analysis.

### Enterocyte isolation

Enterocytes were isolated from sections 2 and 3 of the small intestine as previously described [12, 15]. Briefly, the intestinal sections were washed in tissue buffer (Hank's Balanced Salt Solution with 25 mM HEPES and 1% fetal calf serum) and then placed in isolation buffer (Calcium and magnesium free Hank's Balanced Salt Solution with 1.5mM EDTA). The intestine segments were incubated for 15 minutes at 37°C with rotation. The sample was vortexed briefly and the supernatant containing enterocytes was removed and saved. The process was repeated and the supernatants containing isolated enterocytes were combined.

### CLD isolation

CLDs were isolated from enterocytes using a previously established sucrose gradient ultracentrifugation protocol [16]. Enterocytes were lysed in ice cold sucrose lysis buffer (175mM sucrose, 10 mM HEPES and 1 mM EDTA pH 7.4). Cells were disrupted by passing through a 27 gauge, 1 inch needle, eight times. The resulting two mLs of cell lysate were carefully layered with six mLs of sucrose-free lysis buffer and centrifuged at 20,000 x g at 4°C for two hours. After centrifugation, the sample was frozen at -80°C. The frozen sample was sliced into seven sequential fractions which were approximately 1 cm in length.

### TAG and protein analysis

TAG concentration of each fraction was determined using an L Type TG M assay (Wako Diagnostics, Richmond VA, USA). Protein concentration of each fraction was determined by a BCA assay (Thermo Scientific, Rockford IL, USA). Additionally, a Western Blot analysis was performed on each fraction. Briefly, samples from the isolated fractions were delipidated using 10% sodium dodecyl sulfate, separated by polyacrylamide gel electrophoresis, and transferred to a PVDF membrane. The membranes were probed overnight at 4°C with antibodies for Plin3 (a gift from Dr. Perry Bickel at the University of Texas Southwestern, Dallas TX, USA), glyceraldehyde 3-phosphate dehydrogenase (Gapdh) (Abcam, Cambridge MA, USA), or calnexin (Cnx) (Santa Cruz Biotechnology, Dallas TX, USA). These proteins were used as markers for the CLD, cytosolic, or membrane fractions, respectively. The blots were then probed with a LI-COR IR DYE 800CW secondary antibody and imaged using Odyssey CLx Infrared imaging system (LI-COR Biosciences, Lincoln NE, USA).

### In solution digestion and LC MS/MS

In preparation for proteomic analysis, the isolated CLD fractions were delipidated using 2:1 chloroform methanol and proteins precipitated using ice cold acetone. The protein pellet was denatured using 8M urea and 10 mM DTT for 1.5 hours at 37°C. The sample was digested for 12 hours using trypsin (Sigma-Aldrich, St. Louis MO, USA) using a ratio of 1 μg trypsin to 50 μg protein. The reaction was quenched using trifluoroacetic acid. Tryptic peptides were separated on a nanoLC system (1100 Series LC, Agilent Technologies, Santa Clara, CA). The peptides were loaded on the Agilent 300SB-C18 enrichment column for concentration and the enrichment column was switched into the nano-flow path after five minutes. Peptides were separated with a C18 reversed phase ZORBAX 300SB-C18 column. The column was connected to the emission tip and coupled to the nano-electrospray ionization (ESI) source of the high resolution hybrid ion trap mass spectrometer LTQ-Orbitrap LX (Thermo Fisher Scientific, Waltham MA, USA). The LTQ-orbitrap mass spectrometer was operated in the data-dependent positive acquisition mode in which each full MS scan (30.000 resolving power) was followed by six MS/MS scans where the six most abundant molecular ions were selected and fragmented by collision induced dissociation (CID) using a normalized collision energy of 35%.

### Data analysis and bioinformatics

The peak list files containing MS and MS/MS data were analyzed using MaxQuant version 1.4.08[17]. For protein identification, the MS/MS data was searched against the Uniprot protein database which includes the Swissprot (manually annotated and reviewed) and TrEMBL (automatically annotated and not reviewed) databases [18]. The database was searched using the MASCOT search engine utilizing Andromeda as the peptide search algorithm that is incorporated in the MaxQuant platform [19]. The search was conducted using the following settings: trypsin cleavage with a maximum of two missed cleavages, fixed modification of iodoethanol addition to cysteine, variable modification of oxidation of methionine. The MS mass tolerance was set at 4.5 ppm with a maximum number of five modifications. The false discovery rate was set at 0.01 for proteins and peptides and was run against a decoy revert database. Peptides required a minimum length of seven amino acids. The MS/MS tolerance was set at 0.1 Da for protein identification. The minimum score for modified and unmodified peptides was set at forty. At least two peptides were required for protein identification.

The bioinformatics and statistical package Perseus 1.4.1.3 was used to analyze the MaxQuant output. Contaminants identified by Perseus, such as keratin, were removed from the analysis. For label free quantitation (LFQ), the intensity for each protein was transformed log2(x) and samples who had a peptide intensity below the level of detection were assigned a value of 14. We limited the analysis to proteins which were identified in at least 3 out of the 4 biological replicates.

Proteins identified were clustered by their Gene Ontology (GO) Term based on biological process or molecular function using the Database for Annotation, Visualization and Integrated Discovery (DAVID) v 6.7 [20, 21]. Visualization of protein interactions was accomplished using STRING version 9.1[22]. We used the confidence view with a score of 0.4, indicating a medium confidence level. We did not limit the number of interactions in the analysis.

### Immunofluorescence imaging

Four, C57BL/6 male mice were fasted for 4 hours at the beginning of the light cycle and then administered a 200 μl olive oil bolus. A small (5mm) section of the jejunum was harvested from the mice two hours after the dietary fat challenge and was frozen in optimal cutting temperature embedding media. The tissue was stored at -80°C until processed by immunofluorescence microscopy. The tissue was sliced into 10 μm tissue sections, fixed in 2% paraformaldehyde, and permeabilized with 0.1% saponin. The tissue was probed with previously validated antibodies for Plin3 [23] (a gift from Dr. Perry Bickel at the University of Texas Southwestern, Dallas TX, USA), apolipoprotein A-IV (ApoA-IV) [24] (a gift from Dr. Patrick Tso from the University of Cincinnati, Reading OH, USA), and acyl-CoA synthetase long-chain family member 5 (Acsl5) (personal communication with A. Greenberg) (a gift from Dr. Andrew Greenberg from Tufts University, Boston MA, USA). The sections were also stained for neutral lipids using 1 μg/ml 4,4-difluoro-1,3,5,7,8-pentamethyl-4-bora-3a,4a-diaza-s-indacene (BODIPY) (Life technologies, Grand Island NY, USA), a secondary AlexaFluor antibody (Life technologies), and for nuclei using 300 nM DAPI (Life technologies). The sections were imaged using a Nikon A1R confocal microscope (Nikon Instruments Inc., Melville NY, USA). Image processing, co-localization analysis, and multidimensional imaging were conducted using NIS- Elements C acquisition and analysis software.

### Transmission Electron Microscopy

#### Cardiac perfusion fixation and TEM imaging of jejunum

Two, C57BL/6 mice were fasted for 4 hours at the beginning of the light cycle. An oral gavage of 200 μl olive oil was administered and two hours after the oil bolus, the mice were anesthetized using inhaled isoflurane and perfused with 1.5% glutaraldehyde in 0.1M sodium cacodylate via cardiac infusion. After fixation, a sample of the jejunum section of the small intestine was isolated, stained with osmium tetroxide, dehydrated, and embedded in resin. Ultrathin sections were stained with lead citrate and uranyl acetate and examined using a Tecnai T20 Transmission Electron Microscope (FEI, Hillsboro OR, USA).

#### TEM imaging of isolated CLDs

A sample from the isolated CLD fraction was negatively stained using 2% w/v aqueous uranyl acetate. The isolated CLDs were imaged using a Tecnai T20 Transmission Electron Microscope (FEI).

#### Immunoelectron microscopy

Two, C57BL/6 mice were fasted for 4 hours at the beginning of the light cycle. An oral gavage of 200 μl olive oil was administered and two hours after the oil bolus, the mice were anesthetized using inhaled isoflurane and perfused with 4% paraformaldehyde and 0.05% glutaraldehyde in 0.1M phosphate buffer via cardiac infusion. After fixation, a sample of the jejunum section of the small intestine was isolated, dehydrated, and embedded in resin. Ultrathin sections were probed with an antibody for Acsl5 (a gift from Dr. Andy Greenberg), gold conjugated secondary antibody (Ted Pella Inc. Redding, CA), and stained with uranyl acetate. The samples were examined using Tecnai T20 Transmission Electron Microscope (FEI).

## Results

### Lipid accumulates in CLDs in enterocytes in response to a dietary fat challenge

**Fig 1:**
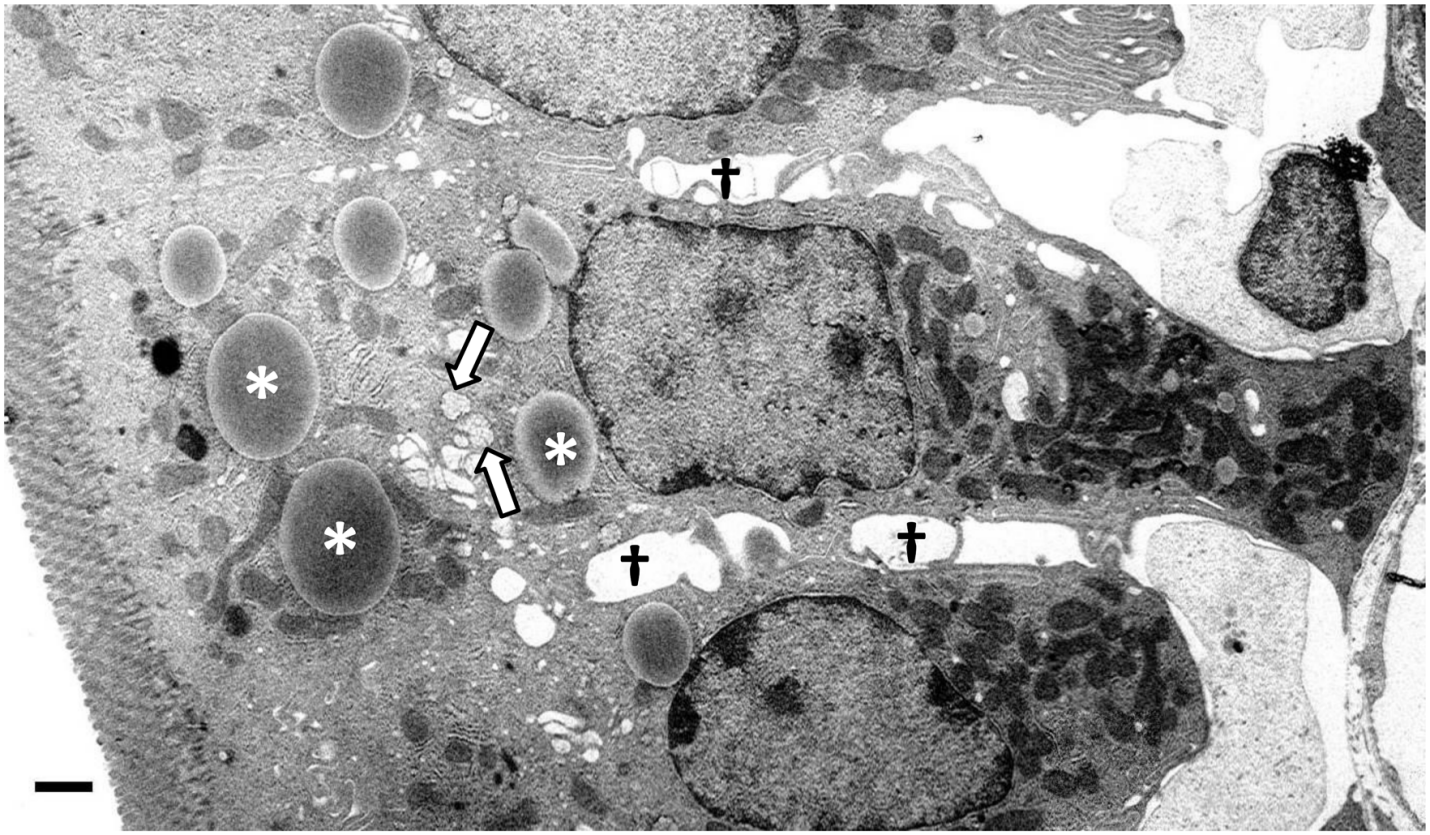
Lipid accumulates in CLDs in enterocytes in response to a dietary fat challenge. Representative transmission electron micrograph of a mouse enterocyte from the jejunum section of the small intestine, two hours after an 200 μl olive oil bolus. Neutral lipids, stained with osmium tetroxide, accumulate in CLDs indicated by white asterisks. Golgi apparatus (white arrows) contain smaller chylomicron sized particles. White areas between enterocytes contain secreted chylomicrons (black cross). Scale bar, 1 μm.

Neutral lipids accumulate in distinct regions of the enterocyte following an acute olive oil bolus. Enterocytes from the jejunum section of the small intestine, two hours after a dietary fat challenge, were stained with osmium tetroxide and examined by transmission electron microscopy (Fig 1). The various fates of neutral lipids within enterocytes following a dietary fat challenge are identified in this image. Neutral lipids accumulate in large CLDs which are indicated by white asterisks. Also visible is the Golgi apparatus (white arrows) which contains smaller chylomicron sized particles. Additionally, the white areas between enterocytes contain secreted chylomicrons (black cross). These results highlight the complex, multistep process of dietary fat absorption.

### The CLD fraction is enriched with Plin3 and has a high TAG to protein ratio

**Fig 2:**
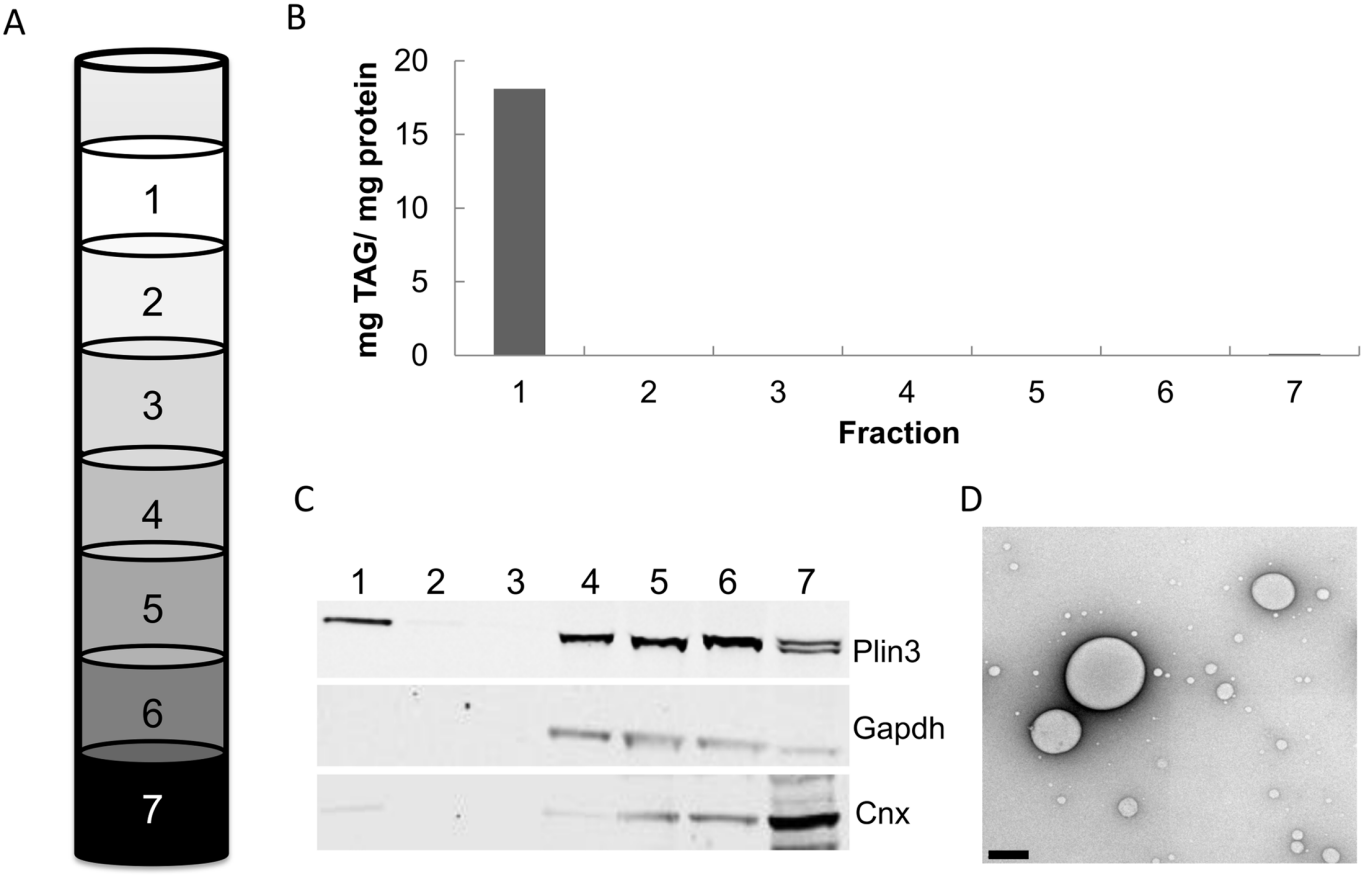
Isolated CLD fraction is enriched with CLD marker, Plin3, and has a high TAG to protein ratio. Enterocytes were isolated from the jejunum section of mouse small intestine, two hours after a 200 μl olive oil bolus. (A) Cells were lysed and fractionated using sucrose gradient ultracentrifugation. After freezing, the sample was cut into 7 fractions. (B) TAG/ protein ratio of isolated fractions. (C) Immunoblot analysis of the fractions with known markers of CLDs (Plin3), cytosol (Gapdh), and membranes (Cnx). (D) Negative staining transmission electron micrograph of fraction 1. Scale bar, 0.5 μm.

Enterocyte CLDs were isolated via density gradient ultracentrifugation using a previously established protocol [16]. After centrifugation, the samples were frozen and sliced into seven fractions, where fraction 1 contains CLDs (Fig 2a). To confirm the successful isolation of CLDs, the isolated fractions were subjected to Western blot analysis. Antibodies for Plin3, Gapdh, and Cnx, were used as markers of the CLD, cytosolic, and membrane fractions respectively. The CLD marker Plin3 was found in fractions 1, 4, 5, 6, and 7 (Fig 2c), representing the various fractions Plin3 has been previously described to locate [25]. Plin3 is found primarily in the cytosol when lipids are absent and translocate to the nascent CLD in the early stages of CLD formation. Gapdh was used as a cytosolic marker and was found primarily in the cytosolic fractions (fractions 2–6). In addition, Gapdh was notably absent in fraction 1. Cnx was used as a marker for membranes and found primarily in fraction 7. To validate the presence of CLDs, the TAG to protein ratio of each fraction was examined (Fig 2b). Fraction 1 contained a high concentration of TAG compared to protein, indicating the presence of CLDs. Fraction 1 was also examined using negative staining electron microscopy which showed an abundance of round, CLD sized particles (Fig 2d). This data suggests that CLDs were successfully isolated from mouse enterocytes.

### Proteins were identified in the CLD fraction through LC MS/MS

**Fig 3:**
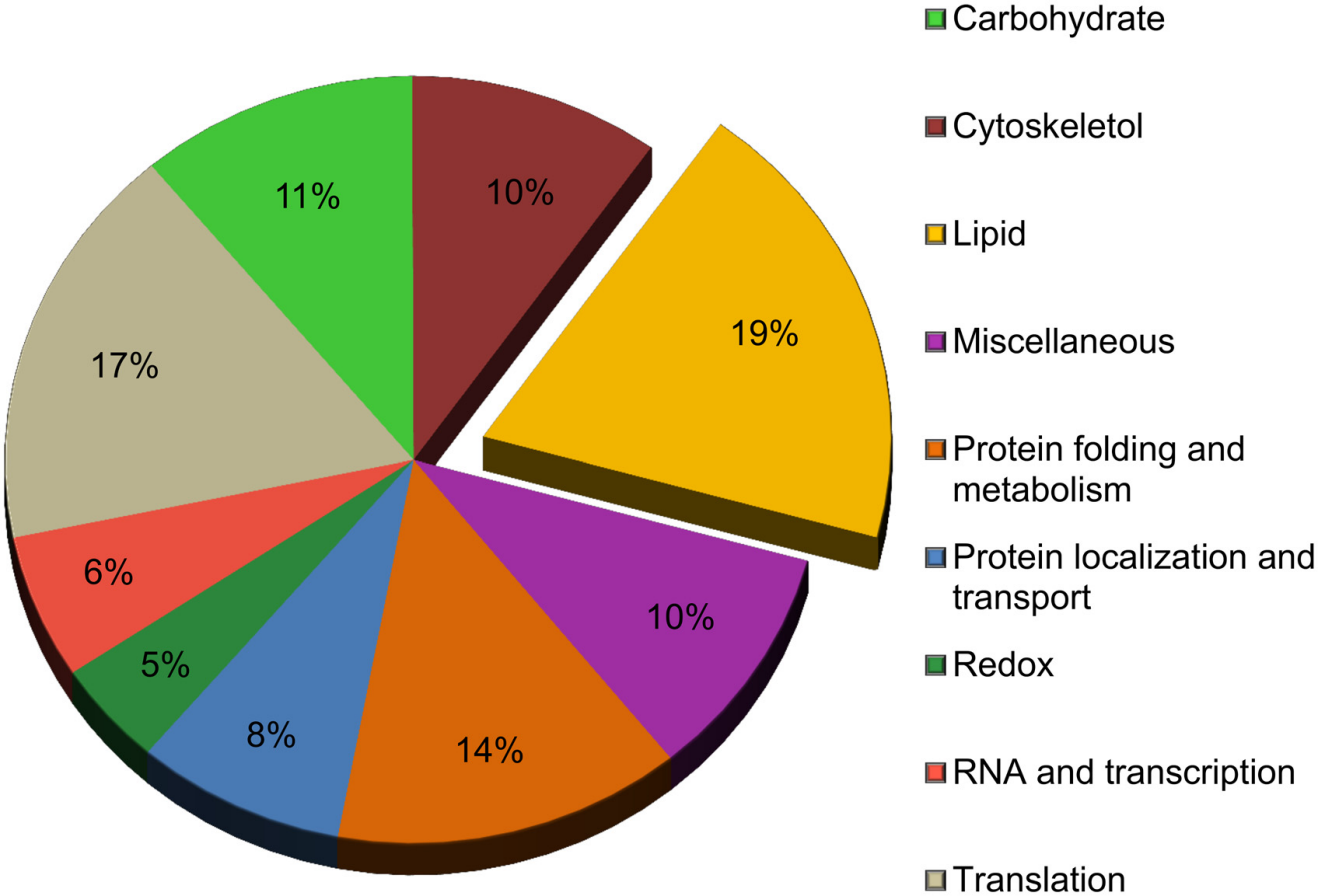
Proteins identified by LC MS/MS and grouped by biological process and molecular function. Identified proteins were assigned to a general classification based on the gene ontology (GO) term for molecular function or biological process using DAVID.

To identify proteins that associate with the CLD fraction, we utilized a shotgun proteomic approach. Through high resolution tandem mass spectroscopy, we identified 181 proteins in at least 3 out of the 4 biological replicates (S1 Table). Proteins were identified by comparing the MS/MS spectra against the Uniprot database. The Uniprot database combines FASTA files from the Swissprot and TrEMBL databases. Using Maxquant’s LFQ [26], the relative abundance of each protein within each sample was identified. It should be noted that the LFQ intensity is the result of multiple factors, including the abundance of the protein within the sample and the ability of the peptides to be ionized and detected. To identify the function of the proteins, we used the functional annotation tool in DAVID. The Gene Ontology (GO) terms of molecular function and biological process associated with each protein was identified and then GO terms were clustered into broader classifications as described in Table 1. The percentage of proteins categorized within broader classifications is found in Fig 3. On several occasions, the GO terms associated with a protein would fall under multiple broader classifications. Under these circumstances, a manual analysis of the protein’s function was conducted using the Uniprot and NCBI databases. Based on the quantity and quality of reported protein function, the protein was assigned into the most appropriate GO term and broader classification.

**Table 1:** GO Terms associated with protein classification term.

Classification	GO_id	GO Term	Number of proteins
Translation	GO:0006412	Translation	22
	GO:0006417	regulation of translation	5
Carbohydrate	GO:0044275	cellular carbohydrate catabolic process	8
	GO:0016052	carbohydrate catabolic process	11
	GO:0044262	cellular carbohydrate metabolic process	11
	GO:0030246	carbohydrate binding	9
	GO:0016051	carbohydrate biosynthetic process	6
Cytoskeletal	GO:0030029	actin filament-based process	12
	GO:0051015	actin filament binding	5
	GO:0008092	cytoskeletal protein binding	11
Lipid	GO:0006629	lipid metabolic process	21
	GO:0044255	cellular lipid metabolic process	18
	GO:0008289	lipid binding	17
	GO:0019216	regulation of lipid metabolic process	10
	GO:0016042	lipid catabolic process	7
	GO:0005811	lipid particle	6
	GO:0016788	hydrolase activity, acting on ester bonds	10
Protein localization and transport	GO:0008104	protein localization	18
	GO:0032880	regulation of protein localization	6
	GO:0015031	protein transport	15
Protein folding and metabolism	GO:0019538	protein metabolic process	47
	GO:0071822	protein complex subunit organization	23
	GO:0042803	protein homodimerization activity	19
	GO:0006461	protein complex assembly	18
	GO:0032403	protein complex binding	17
	GO:0051246	regulation of protein metabolic process	16
	GO:0022613	ribonucleoprotein complex biogenesis	12
RNA and transcription	GO:0051252	regulation of RNA metabolic process	17
	GO:0006403	RNA localization	4
	GO:2001141	regulation of RNA biosynthetic process	15
	GO:0006396	RNA processing	14
Redox	GO:0055114	oxidation-reduction process	27
	GO:0045454	cell redox homeostasis	4
	GO:0051920	peroxiredoxin activity	3

GO terms of biological process and molecular function were grouped under broader classifications. Proteins were assigned into a broader classifications based on the associated GO terms.

### Some identified proteins in the CLD fraction are associated with known lipid metabolism related functions

**Fig 4:**
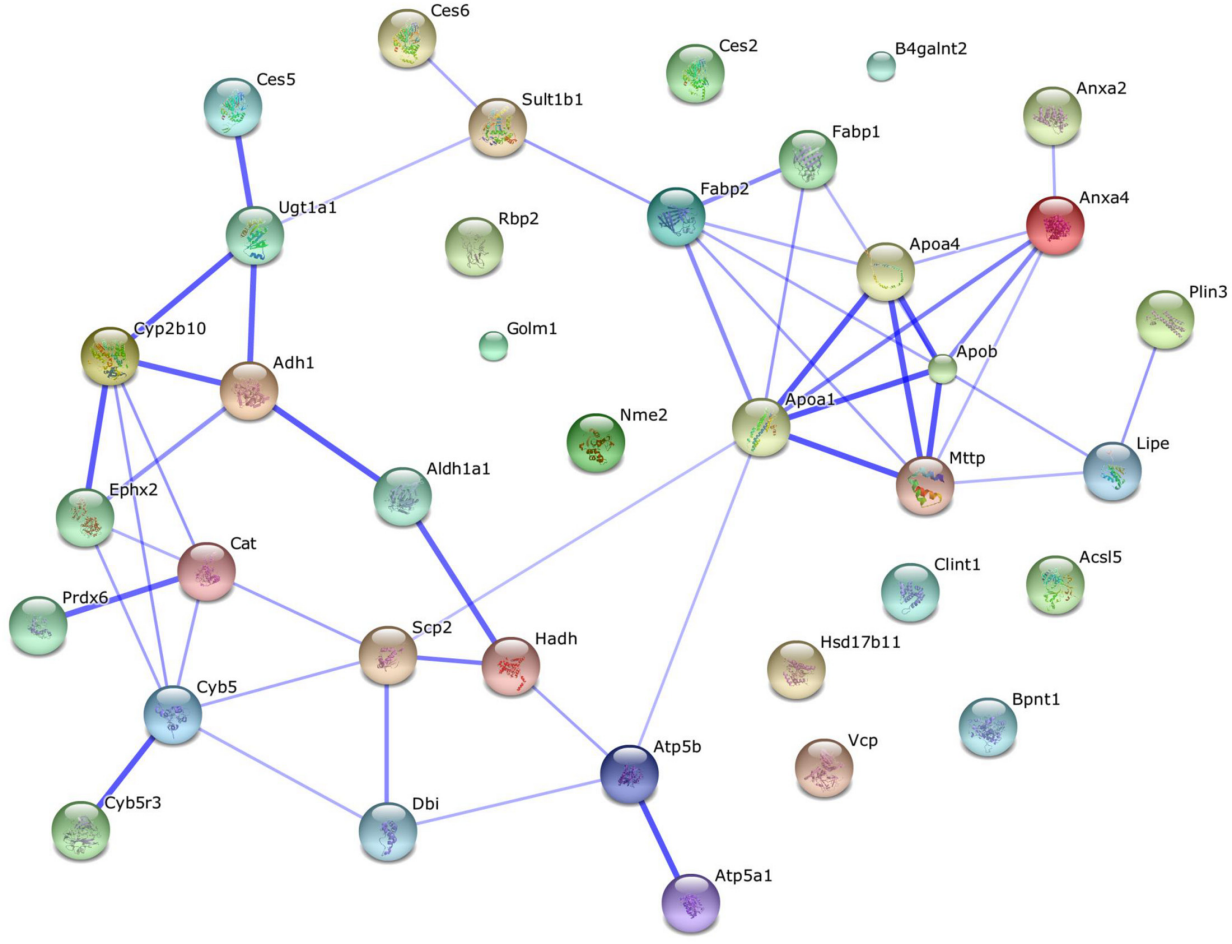
Lipid related proteins identified cluster based on predicted physical and functional interactions. A STRING analysis was conducted to map predicted interactions of lipid related proteins. The resulting network shows several clusters, including proteins responsible for lipoprotein synthesis and modifiers of fatty acids.

Using DAVID’s functional analysis tool, 19% of the proteins identified in the CLD fraction were found to be associated with a biological process or molecular function related to lipid metabolism. The relative abundance of these proteins was determined by Maxquant’s LFQ (Table 2). Approximately half of these proteins have been identified in other CLD proteomic studies from diverse cell types (Table 2). We specifically highlighted proteins which have been previously identified associated with CLDs in an intestinal cell model through proteomic analysis. A map of predicted interactions was generated using a STRING analysis (Fig 4) [22]. The resulting network shows several protein clusters including proteins responsible for lipoprotein synthesis and modifiers of fatty acids.

**Table 2:** A sub group of proteins are associated with known lipid related functions.

Avg LFQ	Protein name	Gene name	Uniprot IDs	Identified in other CLD studies	Identified in intestine model
21.801	Long-chain-fatty-acid—CoA ligase 5	Acsl5	Q8JZR0		[34]
20.926	Alcohol dehydrogenase 1	Adh1	Q3UKA4	[45, 54, 55, 58]	
20.006	Retinal dehydrogenase 1	Aldh1a1	P24549	[45]	
21.008	Annexin A2	Anxa2	P07356	[46, 47, 55, 58, 63]	[33]
21.993	Annexin A4	Anxa4	Q7TMN7	[47]	[33]
23.367	Apolipoprotein A-I	Apoa1	Q3V2G1	[45–47]	
24.529	Apolipoprotein A-IV	Apoa4	Q9DBN0		[33]
18.634	Apolipoprotein B	Apob	E9Q414		
19.522	ATP synthase subunit alpha, mitochondrial	Atp5a1	Q03265	[45, 47]	
22.053	ATP synthase subunit beta, mitochondrial	Atp5b	P56480	[45, 47]	
18.834	Beta-1,4 N-acetylgalactosaminyltransferase 2	B4galnt2	Q09199		
19.963	3(2),5-bisphosphate nucleotidase 1	Bpnt1	Q9Z0S1		
19.926	Catalase	Cat	Q8C6E3	[45, 64]	
18.289	Carboxylesterase 2a	Ces2a	Q8QZR3		
20.492	Carboxylesterase 2c	Ces2c	Q91WG0		
23.281	Carboxylesterase 2e	Ces2e	Q8BK48		
16.681	Clathrin interactor 1	Clint1	Q5SUH7		
22.123	Cytochrome b5	Cyb5a	P56395	[45, 47]	
22.653	NADH-cytochrome b5 reductase 3	Cyb5r3	Q9DCN2	[45]	[33, 34]
20.515	Cytochrome P450 2B10	Cyp2b10	Q9WUD0		
21.318	Acyl-CoA-binding protein	Dbi	Q548W7	[47]	
19.61	Bifunctional epoxide hydrolase 2	Ephx2	Q3UQ71	[45]	
23.106	Fatty acid-binding protein, liver	Fabp1	Q3V2F7	[45]	
22.526	Fatty acid-binding protein, intestinal	Fabp2	Q53YP5		
18.818	Golgi membrane protein 1	Golm1	Q91XA2		
18.524	Hydroxyacyl-coenzyme A dehydrogenase	Hadh	Q61425	[46]	
21.474	Estradiol 17-beta-dehydrogenase 11	Hsd17b11	Q9EQ06	[45, 46]	[33, 34]
18.527	Hormone-sensitive lipase	Lipe	P54310	[46, 47, 56, 57]	[33, 34]
23.765	Microsomal triglyceride transfer protein	Mttp	O08601		[33, 34]
20.812	Nucleoside diphosphate kinase	Nme2	Q01768		
25.467	Perilipin-3	Plin3	Q9DBG5	[46, 56, 58, 64–66]	[33, 34]
19.272	Peroxiredoxin-6	Prdx6	Q6GT24	[45]	
18.859	Retinol-binding protein 2	Rbp2	Q08652		
20.936	Non-specific lipid-transfer protein	Scp2	P32020	[45, 46]	
19.562	Sulfotransferase family cytosolic 1B member 1	Sult1b1	Q9QWG7		
21.097	UDP-glucuronosyltransferase 1-7C	Ugt1a7c	Q6ZQM8		
18.177	Transitional endoplasmic reticulum ATPase	Vcp	Q8BNF8	[45–47]	

Thirty seven proteins associated with known lipid metabolism pathways were identified, of which twenty three proteins have been previously identified in other CLD proteomic analyses. Relative levels of the proteins were determined by LFQ and the average is reported (n = 4 mice).

### Immunofluorescence imaging of proteins identified in the CLD fraction supports their CLD localization

**Fig 5:**
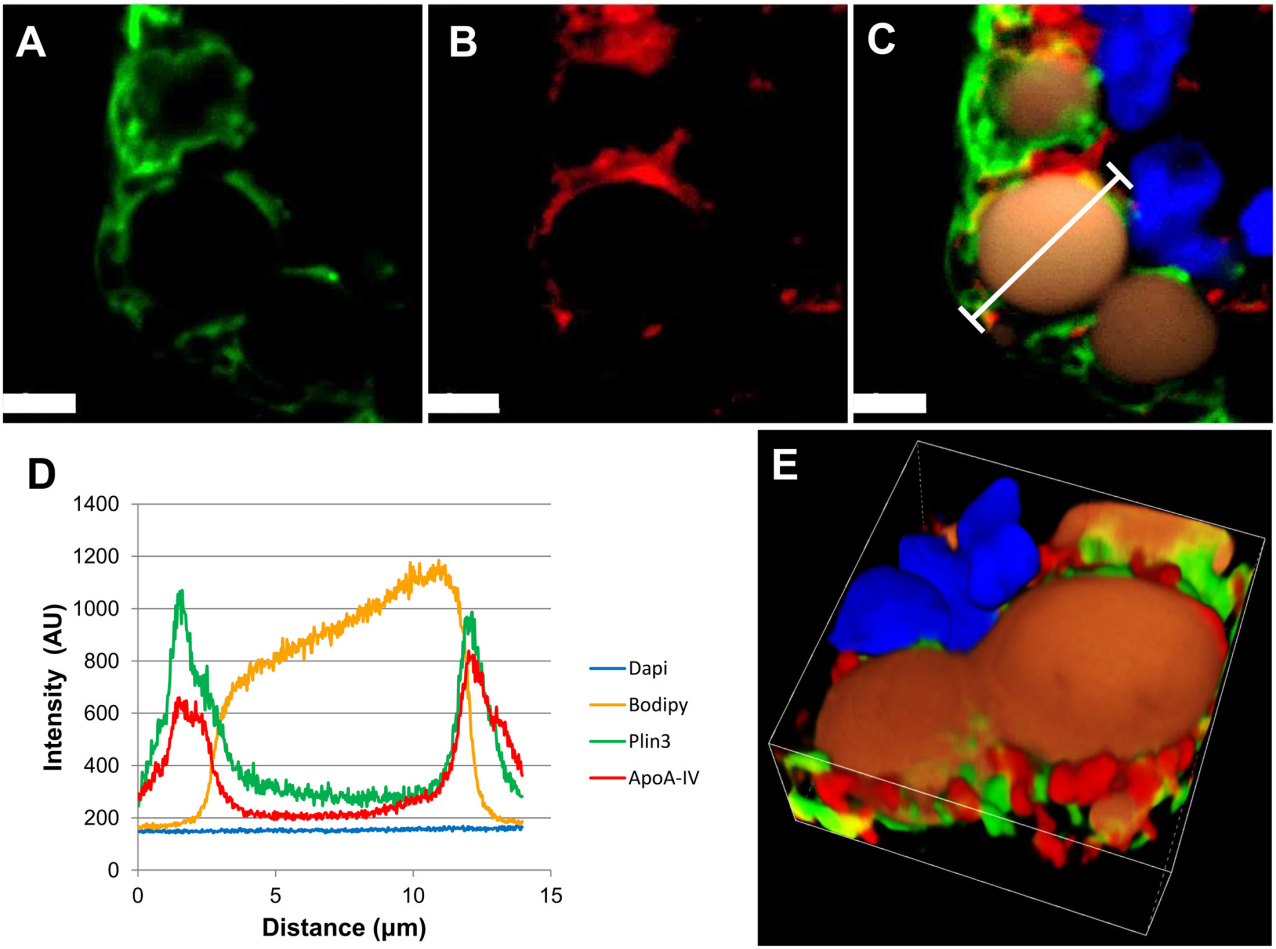
Immunofluorescence imaging of Plin3 and ApoA-IV demonstrates localization on or around CLDs. Representative confocal immunofluorescence images of enterocytes two hours after a 200 μl olive oil bolus (n = 4 mice). Frozen sections were immunostained for Plin3 in green (A) and ApoA-IV in red (B). Lipids were stained with Bodipy (orange) and nuclei stained with Dapi (blue) and the signals were merged. Bars = 5 μm(C). The signals from Plin3 and ApoA-IV have a Pearson’s correlation of 0.59 and a Mander’s overlap of 0.96 indicating a high degree of colocalization. A capped white line denotes a region of interest and the intensity profile along the line of the signals was generated (D) and indicates the signals of Plin3 and ApoA-IV overlap and flank the CLD. A 3D volume view was generated from Z-series images (E) with the dimensions of 15.91 x 15.91 x 7.5 μm was generated and indicates Plin3 and ApoA-IV localizes to the area on or around the CLD.

To confirm the localization of ApoA-IV and Acsl5 on or around CLDs, we probed intestinal sections with antibodies for these proteins from four, male C57BL/6 mice, two hours after a 200 μl olive oil bolus. To visualize CLDs, we stained neutral lipids with BODIPY (Life technologies) and used immunofluorescence staining of a well-established marker of enterocyte CLDs, Plin3 [12]. Due to the process of fixation, neutral lipids are often depleted from CLDs; therefore, where applicable, both Plin3 and neutral lipid staining was used to enhance our ability to detect CLDs. The first protein investigated was ApoA-IV, a protein known regulate intestinal lipid absorption and chylomicron synthesis [27, 28]. To confirm the localization of ApoA-IV to the area on or around the CLD, the signals from Plin3 (Fig 5A) and ApoA-IV (Fig 5B) were merged and overlaid with signals from nuclei (blue) and neutral lipid (orange) (Fig 5C). A region of interest was highlighted (white capped line) in Fig 5C. The intensity profile of all fluorescent signals along this line was generated (Fig 5D). The intensity signals from Plin3 and ApoA-IV overlap and form two peaks which flank the signal from neutral lipids. Additionally, the signal from ApoA-IV and Plin3 antibodies colocalize with a Pearson’s coefficient of r = 0.59 and a Mander’s overlap of r = 0.96 which indicates a high degree of correlation. This indicates that ApoA-IV localizes to the area on or around the CLD. A three dimensional volume view was generated from Z-series images (Fig 5E) and illustrates the localization of Plin3 and ApoA-IV to the area on or around the CLD forming a protein coat. The intensity profile, colocalization analysis, and volume view was generated using NIS- Elements C software.

**Fig 6:**
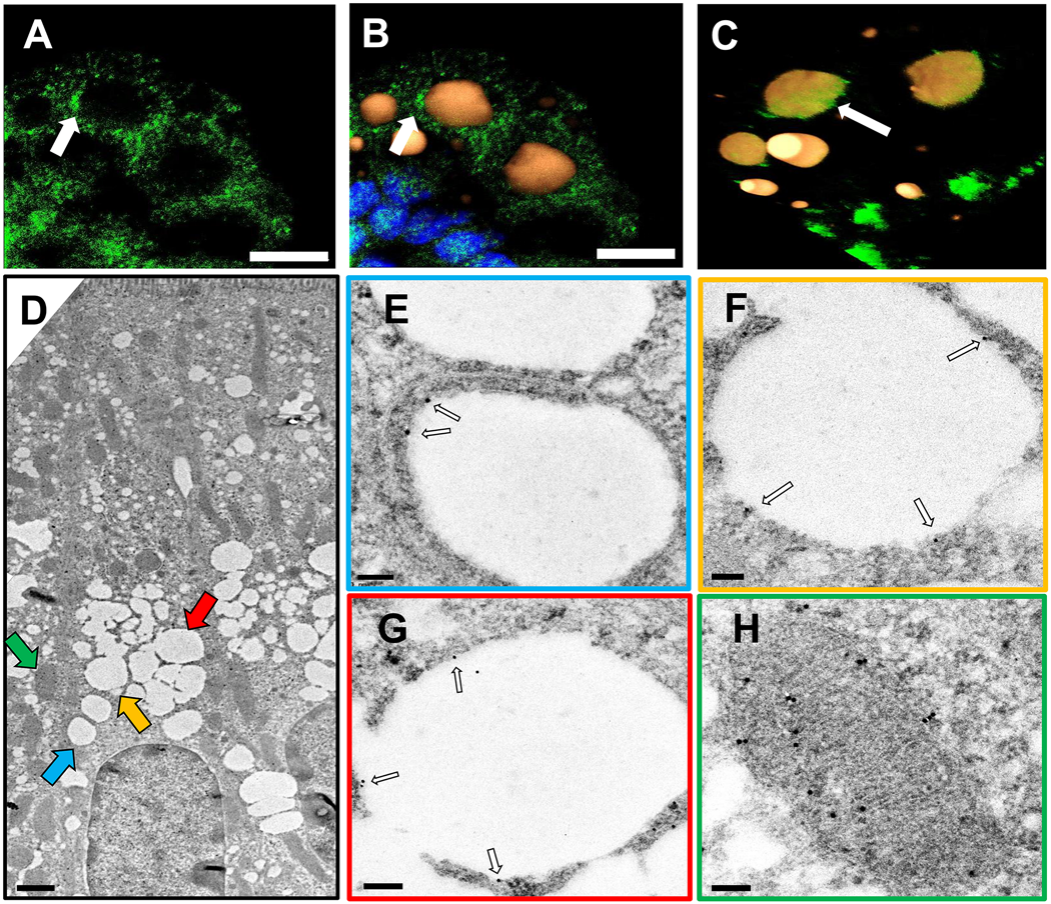
Immunofluoresence and immunoelectron imaging of Acsl5 demonstrates localization on or around CLDs. Representative confocal immunofluorescence images (n = 4 mice) and immunoelectron micrographs (n = 2 mice) of enterocytes two hours after a 200 μl olive oil bolus. Frozen sections were immunostained for Acsl5 (A). Lipids were stained with Bodipy (orange), nuclei stained with Dapi (blue), and the signals were merged. Bars = 5 μm (B). A 3D volume view was generated from Z-series images with the dimensions of 21.21 x 21.21 x 3.25 μm and indicates Acsl5 localizes to the area on or around the CLD (C). An immunoelectron micrograph of an enterocyte containing CLDs two hours post a 200 μl olive oil bolus with areas of interest highlighted by colored arrows. Scale bar = 1 μm. Colored arrows correspond with colored boxes of images E-H (D). CLDs are labeled with nanogold conjugated anti-Acsl5 antibody as indicated by the white arrows. Bars = 100 nm (E-G). Additionally, Acsl5 gold labeling was also observed in mitochondria. Bars = 100 nm (H).

The proteomic analysis of isolated CLDs also identified Acsl5, an enzyme that activates fatty acids by the addition of coenzyme A [29]. In this experiment, we were unable to use Plin3 as a marker for CLD due to the fact the antibodies for Acsl5 and Plin3 were generated in the same host species, so CLDs are identified using only BODIPY. Intestinal sections were probed with an antibody for Acsl5 (green) (Fig 6a), stained for neutral lipids using BODIPY (orange) and nuclei using DAPI (blue) (Fig 6B). Acsl5 forms distinct ring like structures on or around CLDs as indicated by the white arrows. A 3D volume view was generated from Z series images (Fig 6C) showing the localization of Acsl5 to the area on or around CLDs. Additionally, to confirm the localization of Acsl5 to CLDs in enterocytes following a 200 μl olive oil bolus, we performed immunogold labeling of ultra-thin sections of the jejunum section of the small intestine. An electromicrograph (Fig 6D) shows an enterocyte with large white CLDs present. Areas of interest are highlighted by colored arrows which correspond with Fig 6E–6H. Acsl5 conjugated gold particles are found on the CLD surface which are indicated by the white arrows (Fig 6E–6G). In Fig 6E, Acsl5 can be seen localized to the CLD as opposed to the endoplasmic reticulum membrane which can been seen on the left hand side of the CLD. Additionally, Acsl5 can be found localized to the mitochondria (Fig 6H) which has been previously reported in Caco-2 cells[30, 31] and liver McArdle-RH7777 cells[32].

## Discussion

To increase our understanding of the process of dietary fat absorption, we investigated a key step of this process, the storage of dietary fat in CLDs. Proteins associated with CLDs are likely involved in both the storage and trafficking of fatty acids as well as other cellular processes. Therefore, we analyzed the proteome of CLDs from enterocytes isolated from the jejunum section of the small intestine following an oil bolus using high resolution tandem mass spectroscopy. We identified 181 proteins associated with the CLD fraction (S1 Table) that are associated with a wide range of biological and molecular functions (Fig 3). Several of the identified proteins associated with lipid metabolism have been previously identified in other CLDs proteomic analyses from many cell types, while other identified proteins have not been previously reported (Table 2). Furthermore, we confirmed the localization of select proteins through immunohistochemistry and immunoelectron microscopy (Figs 5 and 6).

Of the 181 proteins identified in the CLD fraction of mouse enterocytes, thirty seven proteins are associated with known lipid related processes. Within this subgroup, twenty three proteins (62%) have been identified in CLD proteomic analyses in various cell types and fourteen proteins (38%) have not been previously reported in CLD proteomic analyses from any cell type (Table 2). Interestingly, three of the CLD proteins, ApoA-IV, Acsl5, and microsomal triglyceride transfer protein (Mttp), have been previously identified in a human cell model of enterocytes, Caco-2 cells, but not other cell types [33, 34]. Therefore, these proteins may be unique to the CLD of enterocytes and serve a function on CLDs in dietary fat absorption not previously recognized. We confirmed the localization of ApoA-IV and Acsl5 on or around CLDs through immunohistochemistry and immunoelectron microscopy analysis of mouse enterocytes after a high fat challenge.

ApoA-IV may play important roles in dietary fat absorption due to its localization on lipoproteins and CLDs [33, 35]. Both lipoproteins and CLDs function as lipid storage vesicles and are surrounded by a phospholipid monolayer and a diverse array of proteins [3, 36]. Many of these proteins regulate synthesis and catabolism of these similar structures. Therefore, the identification of common proteins on lipoproteins and CLDs is not surprising. We observed ApoA-IV forming a distinct ring like structure around CLDs and co-localizing with Plin3, an established CLD protein, through immunofluorescence imaging. Consistent with this result, ApoA-IV was also identified on CLDs in Caco-2 cells via immunoelectron microscopy [33]. Although the precise function of ApoA-IV on lipoproteins is not clear, expression of ApoA-IV regulates lipoprotein synthesis and satiety. When over-expressed, ApoA-IV increases TAG secretion from the liver and intestine, by increasing the amount of lipid packaged on apolipoprotein B (ApoB) containing lipoprotein particles [37–40]. Furthermore, ApoA- IV regulates feeding behavior by trafficking to the brain and serving as a satiety signal [41]. Current knowledge of ApoA-IV’s role in lipoprotein metabolism can be applied to the formation of hypotheses regarding the role of ApoA-IV on CLDs. ApoA-IV may regulate CLD synthesis and/or regulate satiety through sequestration of the protein on the CLD. These hypotheses remain to be tested.

Although the identification of lipoprotein associated proteins in the CLD fraction is not surprising, it is important to consider the potential for contamination of the CLD fraction by lipoproteins. To minimize contamination of the CLD fraction by smaller, lipid containing particles the samples were centrifuged at a relatively low speed (20,000 x g). Smaller lipid containing particles such as lumenal lipid droplets, pre-chylomicron transport vesicles, and chylomicrons, are isolated at much faster centrifugation speeds, 106,000 x g [42], 100,000 x g [43], and 197,000 x g [44], respectively. Despite efforts to minimize lipoprotein contamination, we still identified several proteins which are established for association with lipoproteins including ApoA-IV, ApoB, and Mttp. This observation is consistent with other CLD proteomic analyses from a range of cell types that have also identified lipoprotein associated proteins [33, 45], including cell types not known to produce lipoproteins [46, 47]. Furthermore, ApoA-IV and ApoB were demonstrated to localize to CLDs through immunoelectron microscopy in Caco-2 cells [33] and hepatocytes [48] respectively. Together, these results support that a protein may be associated with both lipoproteins and CLDs.

Acsl5 may play a role in localized TAG synthesis on CLDs in enterocytes as part of the process of dietary fat absorption. It was recently demonstrated in lipid challenged *Drosophilia* S2 cells that TAGs synthesis enzymes localize to CLDs including glycerol-3-phosphate acyltransferase 4, 1-acyl-sn-glycerol-3-phsophate acyltransferase gamma, and diacylglycerol acyltransferase 2 [11]. In the intestine, two TAG synthesis enzymes that catalyze the committed step in TAG biosynthesis, diacylglycerol acyltransferase 1 and diacylglycerol acyltransferase 2 were identified as associated with CLDs by western blotting [49]. Additionally, Acsl enzymes activate fatty acids and channel them towards specific metabolic fates within cells [50]. Interestingly, other Acsl family members including Acsl1 [45, 46], Acsl3 [33, 34, 46], and Acsl4 [33, 46] have been identified on CLDs in various cell types. In particular, Acsl3 over-expression in Cos-1 cells results in generation and expansion of CLDs in response to fatty acids [51]. Finally, Acsl5 directs exogenous fatty acids toward TAG synthesis in hepatocytes [32, 52]. Therefore, one hypothesis for the function of Acsl5 on CLDs in enterocytes is the activation of fatty acids for TAG synthesis; however, this hypothesis remains to be tested.

A limitation to a proteomic approach for identifying CLD associated proteins is the challenge to distinguish protein contamination from other cellular organelles from *bona fide* CLD proteins in the isolated CLD fraction. It is possible that proteins from other organelles associate with the CLD fraction nonspecifically during cell lysis because of the normal tight association of CLDs with other organelles [53]. Methods described in the literature for isolation of CLDs vary in how cells are lysed and the type and numbers of washes used. Currently there is not one commonly accepted method for CLD isolation to alleviate this problem. CLD isolation protocols involving multiple washing steps remove proteins known to associate with other organelles, as well as decrease the levels of well-established CLD proteins [45]. In the current study, we used a single step isolation protocol to preserve potentially loosely bound CLD proteins and reduce the risk of missing key players in CLD metabolism. However, we only included proteins identified in 3 of 4 biological replicates to rule out random contamination. Due to these limitations, each protein identified needs to be validated by other methods such as imaging to confirm the localization to CLDs in enterocytes before making firm conclusions. Despite these limitations, the identification of Acsl5 on CLDs, ER, and mitochondria highlights that a single protein may have multiple associations in the cell. The protein list generated here is intended to be used an informed, hypothesis generating tool to prioritize future research, not a list of established proteins on CLDs in enterocytes.

The proteins identified in the current study build upon an existing body literature of CLD biology, from which interesting commonalities are being observed. A key feature, of all CLD proteomic analyses from multiple cell types, is the identification of non-lipid related proteins in the CLD fraction. These proteins fall into a wide range of biological and molecular functions such as amino acid metabolism, carbohydrate metabolism, and membrane trafficking (Fig 3) [33, 34, 45–47, 54–58]. This consistent observation has not been fully investigated and remains an intriguing paradox of CLD biology. Several hypotheses have been proposed to address this issue including the role of CLDs as sequestration sites [59, 60] or in the regulation of membrane trafficking [61]. The identification of non-lipid related protein in the CLD fraction has expanded our perception of the CLD from a simple lipid storage site to a complex and multi-functional organelle [62].

We successfully isolated CLDs from the jejunum of mice after a dietary fat challenge and identified the proteome of the CLDs. Similar to previously reported CLD proteomic analyses, the proteins identified in the CLD fraction are associated with a wide range of biological processes and molecular functions. Of interest, ApoA-IV and Acsl5 were identified in the proteomic analysis of the CLD fraction and the presence was confirmed through immunofluorescence and immunoelectron microscopy. The role of many of these lipid related proteins on the CLD is currently unknown, however the proteomic analysis provides a foundation to further investigate the role of these proteins and advance the model of dietary fat absorption and lipid trafficking in the small intestine.

## Supporting Information

S1 Table Complete proteome of the CLD fraction isolated from enterocytes. (XLSX)
